# The pathogenic mutations of *APOA5* in Chinese patients with hyperlipidemic acute pancreatitis

**DOI:** 10.1186/s12944-024-02011-5

**Published:** 2024-02-08

**Authors:** Yuxin Liu, Si Dai, Shuqi Qin, Jing Zhou, Zhendan Wang, Guojian Yin

**Affiliations:** 1 Department of Gastroenterology, The Second Affiliated Hospital of Soochow University, District, No.1055, San-Xiang Road, Gu-Su, Suzhou, 215000 Jiangsu Province China; 2 Department of Gastroenterology, Songtao Miao Autonomous County People’s Hospital, Tongren, 554199 Guizhou Province China

**Keywords:** Hyperlipidemic acute pancreatitis, Severe hypertriglyceridemia, Apolipoprotein A-V, Gene mutations

## Abstract

**Background and aims:**

To study the role of gene mutations in the development of severe hypertriglyceridemia (HTG) in patients with hyperlipidemic acute pancreatitis (HLAP), especially different apolipoprotein A5 (*APOA5*) mutations.

**Methods:**

Whole-exome sequencing was performed on 163 patients with HLAP and 30 patients with biliary acute pancreatitis (BAP). The pathogenicity of mutations was then assessed by combining clinical information, predictions of bioinformatics programs, information from multiple gene databases, and residue location and conservation. The pathogenic mutations of *APOA5* were visualized using the software.

**Results:**

1. Compared with BAP patients, pathogenic mutations of *APOA5* were frequent in HLAP patients; among them, the heterozygous mutation of p.G185C was the most common.

2. All six pathogenic mutations of *APOA5* identified in this study (p.S35N, p.D167V, p.G185C, p.K188I, p.R223C, and p.H182fs) were positively correlated with severe HTG; they were all in the important domains of apolipoprotein A-V (apoA-V). Residue 223 is strictly conserved in multiple mammals and is located in the lipoprotein lipase (LPL)-binding domain (Pro215–Phe261). When Arg 223 is mutated to Cys 223, the positive charge of this residue is reduced, which is potentially destructive to the binding function of apoA-V to LPL.

3. Four new *APOA5* mutations were identified, namely c.563A > T, c.667C > T, c.788G > A, and c.544_545 insGGTGC.

**Conclusions:**

The pathogenic mutations of *APOA5* were specific to the patients with HLAP and severe HTG in China, and identifying such mutations had clinical significance in elucidating the etiology and subsequent treatment.

**Graphical Abstract:**

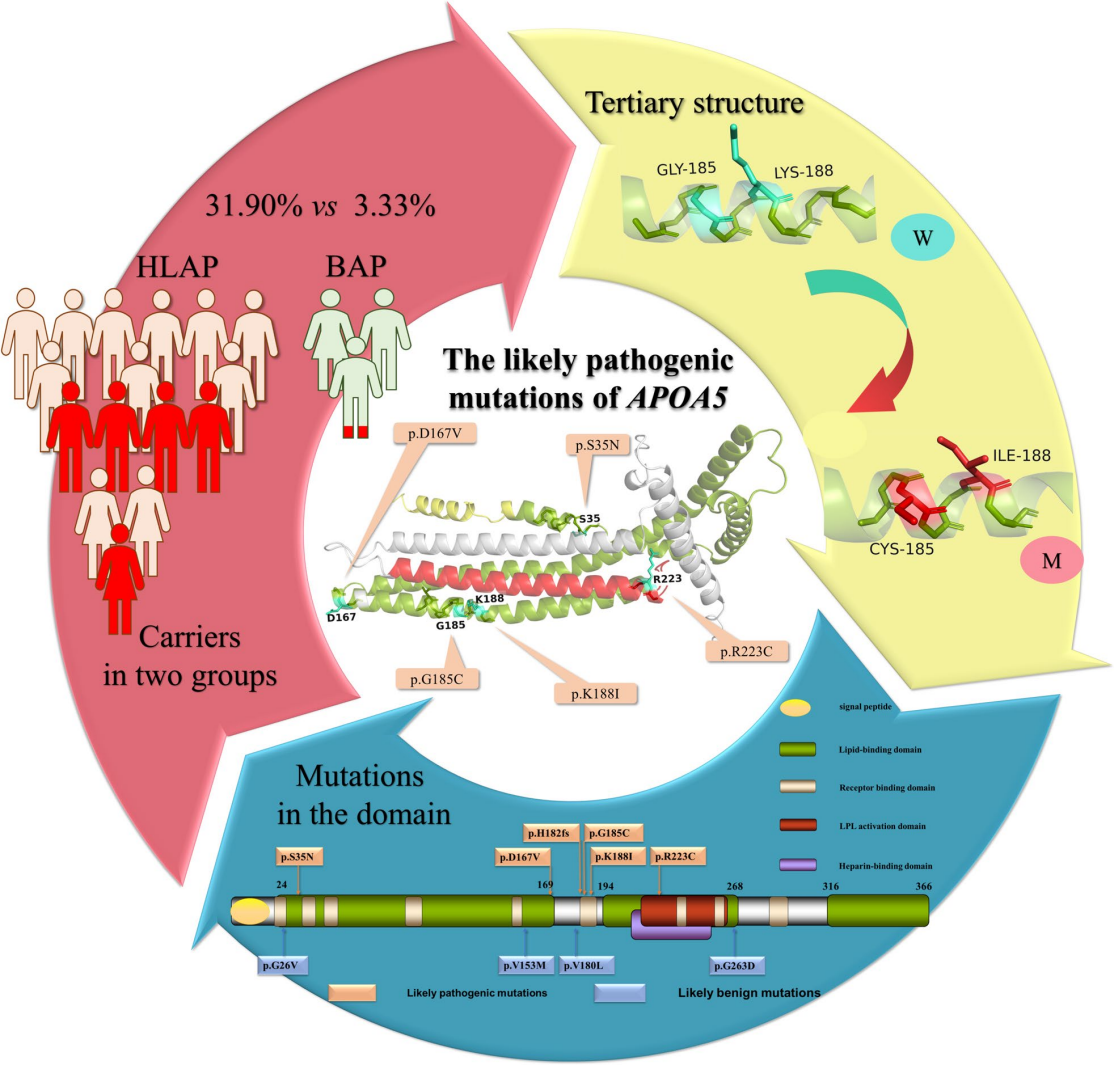

## Introduction

Serum triglyceride (TG) concentrations > 1.7 mmol/L are considered to indicate hypertriglyceridemia (HTG). Hyperlipidemic acute pancreatitis (HLAP) has been reported to occur in approximately 15–20% of adults with severe HTG, meaning TG ≥ 11.3 mmol/L [1]. Currently, the incidence of acute pancreatitis (AP) is rising worldwide [2], and HTG has been reported to surpass alcohol as the second leading cause of AP in some areas of China, such as Beijing and southern Sichuan [3, 4]. According to a previous study, in terms of etiology, biliary acute pancreatitis (BAP) was the most prevalent (53.31%), followed by hyperlipidemic (HLAP) (19.38%), in Suzhou and Shanghai between July 2009 and June 2013 [5]. This phenomenon may have certain connections with the gradual westernization of the Chinese diet. Ye and Leeming found that, in China, average meat consumption increased more than twofold in rural and about 1.5 times in urban areas from 1990 to 2021. From 1991 to 2015, the proportion of energy intake from fat in China increased from 24.0% to 35.8% [6]. This is likely to lead to a yearly increase in the average serum TG levels in the Chinese population [7].

Several recent studies have reached the conclusion that severe HTG is the result of genetic mutations, unhealthy lifestyles, and underlying diseases, among which genetic mutations account for the majority [8–10]. Therefore, the aim of this study was to investigate which genetic mutations may contribute to severe HTG in patients with HLAP.

In this study, pathogenic mutations in apolipoprotein A5 (*APOA5*) were most common in patients with HLAP compared with patients with BAP. *APOA5* is a member of the newly discovered *APOA4* / *APOC3* / *APOA1* apolipoprotein cluster, located on the long arm of chromosome 11, region II, band III (11q23) [11]. Its product is apoA-V, with 366 amino acid residues [12], which is produced mainly in the liver, with plasma concentrations ranging from 20 to 500 ng/mL. It is found mainly in celiac microsomes (CM), very low-density lipoproteins (VLDL), and high-density lipoproteins (HDL). Although apoA-V has low concentrations in the blood, it has been shown to be an important regulator of TG [13]. In addition, *APOA5* polymorphisms show strong geographic and ethnographic variation; for example, c.553 G>T (rs2075291) is prevalent and concentrated in East Asia. In 2004, when *APOA5* had just been recognized, Hubácek *et al.* did not find p.G185C in 83 hyperlipidemic patients and 420 healthy individuals among the Caucasian population [14]. However, Chiou *et al.* and Khovidhunkit *et al.* reported that this variant is very common in Taiwan and Thailand [9, 15]. Many studies have confirmed that multiple polymorphisms of *APOA5* have positive associations with HTG in Chinese people [16–18].

This study is the first to explore the impact of genetic mutations on severe HTG in HLAP patients as compared with BAP patients. Considering that *APOA5* variants are closely related to HTG in Chinese people, the study makes a further step to identify several novel pathogenic mutations in *APOA5* associated with severe HTG.

## Patients and methods

### Participants

The study was approved by the Ethics Committee of the Second Affiliated Hospital of Soochow University (JD-LK-2021-001-01). According to the latest Chinese guidelines for the diagnosis and treatment of AP [19], 163 patients with HLAP and 30 patients with BAP attending the Second Affiliated Hospital of Soochow University from October 24, 2014, to May 31, 2023, were included in this study.

First, all patients needed to fulfill two conditions: (1) Meeting two of the three diagnostic criteria for AP: persistent severe epigastric pain, often radiating to the back; serum lipase (or amylase) at least three times the upper limit of normal; CECT (enhanced computed tomography) or MRI (magnetic resonance imaging) or trans-abdominal ultrasonography with characteristic manifestations of AP [20–24]. (2) Laboratory data available within 48 hours of the onset, including white blood cells (WBC), C-reactive protein (CRP), blood urea nitrogen (BUN), serum calcium (Ca^2+^), blood glucose (GLU), triglyceride (TG), and serum cholesterol (TC). Second, patients with HLAP also needed to show TG ≥ 11.3 mmol/L [25]. Patients with BAP needed to meet the criterion that biliary stones had been found by ultrasound, CT, MRCP (magnetic resonance cholangiopancreatography), or endoscopy [26]. Finally, patients were excluded on the basis of (1) incomplete information, (2) being diagnosed with chronic pancreatitis, and (3) being < 18 years of age.

The severity of AP was classified as mild (MAP), moderately severe (MSAP), and severe AP (SAP) according to the Atlanta Classification of Acute Pancreatitis Revision [27].

### Genetics and data analysis

#### Whole exome sequencing

Blood samples from patients who had fasted overnight were temporarily stored in a refrigerator (BCD-206WECX, Changhong Meiling Co., Anhui, China) at 0°C. Within 24 hours, the blood sample underwent preliminary processing in a centrifuge (TDL-60B; JiaPeng Technology Co., Shanghai, China). The bottom layer of blood cells obtained by centrifugation were stored uniformly in a freezer at -80°C (DW-86L938; IceStar Refrigeration & Electrical Co., Zhejiang, China). The DNA was extracted with the TIANamp Genomic DNA Kit (DP304-02; Tengen Biochemical Technology Co., Beijing, China) and tested for quality with an Ultraviolet Spectrophotometer (NanoDrop 2000; Thermo Fisher Scientific, Shanghai, China), Qubit Fluorometer (Q32857; Life Technologies, Shanghai, China), and agarose gel electrophoresis. The genomic DNA samples that passed were first randomly cut into 150–220 bp fragments using the Covaris Sample Preparation System (S220; Covaris, Woburn, Massachusetts), and the Agilent SureSelect Human All Exon kit (V6; Agilent Technologies, California, USA) was used for gene library construction and capture. The library was tested and qualified for double-end sequencing using a genetic DNA sequencer (NovaSeq6000; Illumina Scientific Equipment Co., Beijing, China). The sequenced raw data was quality controlled using fastp [28] to obtain clean reads. Following this, using BWA [29], the clean reads were compared with the reference genome GRCh37.p13, and the results were converted to SAMtools [30] format, de-redunded using Picard analysis software, and the detected variant information was analyzed using Qualimap software. Finally, single nucleotide variants (SNVs), insertions, and deletions (InDels) were detected using GATK4 [31] on the comparison results (processed by the Shanghai Luminous Biotechnology Company).

#### Multiple databases of *APOA5*-related information

The single nucleotide polymorphism (SNP) numbers of *APOA5* were obtained from the Single Nucleotide Polymorphism Database (dbSNP), which was established by the National Center for Biotechnology Information (NCBI) for storing SNP information for humans and other organisms. The Genome Aggregation Database (gnomAD) is a large database of human variations from whole genome sequencing. Information on included mutations may be found in gnomAD v2.1.1, such as the number and allele frequency in total, East Asian, and Latin American populations. If the variation is not indexed in multiple databases such as the 1000 Genomes Project, the Exome Aggregation Consortium (ExAC), and gnomAD, as well as not having been reported in a paper, it is considered a new discovery.

#### In silico analysis of mutant apoA-V protein functionality

All non-synonymous SNVs were evaluated by six bioinformatics prediction programs: SIFT (http://sift.jcvi.org), PolyPhen (http://genetics.bwh.harvard.edu/pph2), LRT (http://www.genetics.wustl.edu/jflab/lrt_query.html), Mutation Taster (http://mutationtaster.org), Mutation Assessor (http://mutationassessor.org/r3/), and CADD (http://cadd.gs.washington.edu). When two or more of the above six programs judge a mutation to be “damaging or deleterious” (which must include SIFT), that mutation was considered “pathogenic” in this study.

All frameshift mutations (including insertion and deletion), stopgain (newly generated stop codons), and stoploss (stop codon loss) were also classified as “pathogenic”. *APOA5* sequences from 20 mammalian species, mainly primates (data from the UCSC database), were aligned using the phylo-HMM model to assess residue conservation; the higher the score, the more conserved the residue.

The apoA-V sequences of different species (human, rat, mouse, Hawaiian monk seal, cheetah, and harbor seal) were found in the UniProt database. The mutated apoA-V in this study were imported together into MEGA (Molecular Evolutionary Genetics Analysis) software for multiple sequence alignment and finally refined by ESPript 3.0 (https://espript.ibcp.fr/ESPript/cgi-bin/ESPript.cgi). The domains of the wild-type apolipoprotein A-V (apoA-V) were predicted by SignalP-5.0 (https://services.healthtech.dtu.dk/services/SignalP-5.0/), InterPro (http://www.ebi.ac.uk/interpro/), and SMART (http://smart.embl-heidelberg.de/smart/set_mode.cgi?NORMAL=1). The apoA-V tertiary structure was predicted by AlphaFold (https://alphafold.ebi.ac.uk/entry/Q6Q788), and different mutation products were visualized by PyMOL software.

### Statistical analysis

The statistical analyses were performed using SPSS version 25 (IBM, Armonk, NY, USA). All continuous variables were expressed as mean ± standard deviation (SD), and all categorical variables were expressed as numbers and percentages. Comparisons between two groups were made using appropriate tests (all are two-tailed), such as the Student’s *t*-test, Pearson’s *χ*^*2*^ test, Fisher’s exact *χ*^*2*^ test, and the Mann-Whitney U test; *P* values < 0.05 were considered statistically significant.

## Results

### Clinical characterization

The clinical and laboratory characteristics of the 193 patients are summarized in Table 1. The mean age of the patients with HLAP (39.71 ± 9.86 years) was younger (*P* < 0.001) compared with the BAP group (58.3 ± 15.95 years), which is consistent with previous studies in which HLAP patients had a younger age of onset [32]. Patients with HLAP had a higher mean body mass index (BMI) (27.76 ± 3.72 *vs.* 25.11 ± 4.32 kg/m2,* P* = 0.001) and more comorbid fatty liver (138, 85% *vs.* 7, 23%, *P* < 0.001). TG and TC concentrations within 48 hours of onset differed significantly between the two groups (*P* < 0.001). Although there was no difference in hospital stay, it was found that there were more patients in the HLAP group with MSAP and SAP; they had more severe inflammatory reactions and were more prone to recurrence, which is consistent with previous findings [4, 33]. The number of patients who developed acute respiratory distress syndrome (ARDS) in both groups (10, 6% *vs.* 2, 7%) was not significantly different (*P* = 0.912). Patients with HLAP had higher blood glucose (GLU); on the one hand, more of them had comorbid diabetes mellitus (DM), and on the other hand, they had more severe pancreatic inflammation leading to disruption in the pancreatic endocrine system and reduced insulin secretion, which further elevated GLU. There were no statistical differences between the two groups in the comparison of gender, BUN (blood urea nitrogen), creatinine, and blood calcium.

**Table 1:** Clinical characteristics of 193 patients with acute pancreatitis

	HLAP group (*n* = 163)	BAP group (*n* = 30)	*P* value
Age (years)	39.71 ± 9.86	58.3 ± 15.95	< 0.001^***^
Male, *n* (%)	128(80.5%)	11(68.8%)	0.433
Hospital stay (days)	10.47 ± 7.31	10.43 ± 4.64	0.561
BMI (kg/m^2^)	27.76 ± 3.72	25.11 ± 4.32	0.001^**^
WBC (× 10ꝰ /L)	14.14 ± 4.18	11.69 ± 3.84	0.002^**^
CRP (mg/L)	185.08 ± 115.24	61.66 ± 64.39	< 0.001^***^
BUN (mmol/L)	5.76 ± 9.17	4.73 ± 1.84	0.950
Serum creatinine (μmol/L)	70.04 ± 27.18	68.36 ± 13.34	0.551
Ca^2+^ (mmol/L)	2.06 ± 0.23	2.09 ± 0.22	0.256
GLU (mmol/L)	10.68 ± 5.20	7.38 ± 2.70	0.001^**^
TG (mmol/L)	18.00 ± 19.30	0.91 ± 0.34	< 0.001^***^
TC (mmol/L)	9.20 ± 5.27	4.02 ± 0.90	< 0.001^***^
Diabetes mellitus,* n* (%)	87(53%)	4(13%)	< 0.001^***^
Hypertension, *n* (%)	36(22%)	12(40%)	0.037^*^
Fatty liver, *n* (%)	138(85%)	7(23%)	< 0.001^***^
ARDS, number, *n* (%)	10(6%)	2(7%)	0.912
SAP number, *n* (%)	10(6%)	2(7%)	0.912
MSAP & SAP number, *n* (%)	83(50.9%)	5(17%)	0.001^**^
CTSI > 4 score number, *n* (%)	72(44.2%)	5(16.7%)	0.005^**^
Recurrence, *n* (%)	91(55.8%)	7(23.3%)	0.001^**^
Repeated recurrence, *n* (%)	61(37.4%)	3(10.0%)	0.003^**^

*BMI* body mass index, *WBC* white blood cells, *CRP* C-reactive protein, *BUN* blood urea nitrogen, *Ca*^*2*+^ serum calcium, *GLU* blood glucose, *TG* serum triglyceride, *TC* serum cholesterol, *ARDS* acute respiratory distress syndrome, *MSAP* moderately severe acute pancreatitis, *SAP* severe acute pancreatitis, *CTSI* computed tomography severity index, Repeated recurrence: at least two episodes of recurrence in one patient

^*^P < 0.05

^**^P < 0.01

^***^P < 0.001

### Mutation characterization and bioinformatics predictions

#### Comparison of lipid-related mutated genes

This study focused on 53 lipid-related genes to investigate which gene mutations had significant effects on severe HTG in HLAP patients [34–38]. The specifics of the genes carried by the two groups are shown in Table 2. The left half of Table 2 shows the number of patients carrying all benign DNA changes, SNPs, and pathogenic mutations in this gene, and the right half shows only pathogenic mutations. Considering that not all variants cause severe HTG, the rational choice should be to find pathogenic mutations. For example, when focusing on all variants, 163 (100%) of HLAP patients *vs.* 30 (100%) of BAP patients carried variants in the *LIPC* gene. However, when focusing on pathogenic mutations, the number became 8 (5%) *vs.* 0 (0%). The same was true for *APOA5*. When all variants were included, the number of individuals carrying *APOA5* variants in the HLAP group *vs.* the BAP group was 97 (60%) *vs.* 13 (43%), and when focusing on pathogenic mutations, this became 52 (32%) *vs.* 1 (3%).

**Table 2:** All DNA variants and pathogenic mutations identified in the candidate genes of HLAP and BAP patients

Mutant gene	All DNA variants^**a**^		Pathogenic mutations^**b**^	
	HLAP group^**c**^	BAP group	HLAP group	BAP group
*APOA5*	97 (60%)	13 (43%)	52 (32%)	1 (3%)
*PLA2G6*	44 (27%)	7 (23%)	9 (6%)	0 (0%)
*LIPC*	163 (100%)	30 (100%)	8 (5%)	0 (0%)
*LRP1*	163 (100%)	30 (100%)	13 (8%)	1 (3%)
*LMF2*	163 (100%)	30 (100%)	6 (4%)	0 (0%)
*FGR*	7 (4%)	1 (3%)	5 (3%)	0 (0%)
*CETP*	111 (68%)	25 (83%)	15 (9%)	2 (7%)
*PLTP*	21 (13%)	2 (7%)	4 (2%)	0 (0%)
*PINX1*	12 (7%)	4 (13%)	4 (2%)	0 (0%)
*FRMD5*	104 (64%)	14 (47%)	3 (2%)	0 (0%)
*GCKR*	84 (52%)	16 (53%)	3 (2%)	0 (0%)
*IRS1*	102 (63%)	17 (57%)	2 (1%)	0 (0%)
*HAVCR1*	144 (88%)	29 (97%)	2 (1%)	0 (0%)
*SORL1*	163 (100%)	30 (100%)	7 (4%)	1 (3%)
*PPARG*	83 (51%)	8 (27%)	1 (1%)	0 (0%)
*APOBEC1*	141 (87%)	25 (83%)	1 (1%)	0 (0%)
*PPARD*	151 (93%)	27 (90%)	1 (1%)	0 (0%)
*TRIB1*	86 (53%)	15 (50%)	1 (1%)	0 (0%)
*FADS2*	1 (1%)	0 (0%)	1 (1%)	0 (0%)
*FADS3*	2 (1%)	0 (0%)	1 (1%)	0 (0%)
*ANGPTL4*	36 (22%)	7 (23%)	1 (1%)	0 (0%)
*MLXIPL*	162 (99%)	30 (100%)	1 (1%)	0 (0%)
*GALNT2*	153 (94%)	29 (97%)	1 (1%)	0 (0%)
*PPARA*	17 (10%)	4 (13%)	1 (1%)	0 (0%)
*GPIHBP1*	144 (88%)	29 (97%)	1 (1%)	0 (0%)
*TIMD4*	134 (82%)	30 (100%)	1 (1%)	0 (0%)
*USF1*	1 (1%)	0 (0%)	0 (0%)	0 (0%)
*APOC2*	0 (0%)	0 (0%)	0 (0%)	0 (0%)
*MSL2L1*	0 (0%)	0 (0%)	0 (0%)	0 (0%)
*MTP*	0 (0%)	0 (0%)	0 (0%)	0 (0%)
*ZNF664*	3 (2%)	1 (3%)	0 (0%)	0 (0%)
*FADS1*	2 (1%)	1 (3%)	0 (0%)	0 (0%)
*CYP26A1*	1 (1%)	1 (3%)	0 (0%)	0 (0%)
*CILP2*	18 (11%)	5 (17%)	0 (0%)	0 (0%)
*APOC3*	83 (51%)	23 (77%)	0 (0%)	0 (0%)
*CAPN3*	43 (26%)	6 (20%)	5 (3%)	1 (3%)
*NCAN*	92 (56%)	16 (53%)	1 (1%)	1 (3%)
*ANGPTL3*	9 (6%)	1 (3%)	1 (1%)	1 (3%)
*XKR6*	5 (3%)	1 (3%)	1 (1%)	1 (3%)
*LDLR*	155 (95%)	29 (97%)	1 (1%)	1 (3%)
*LDLRAP1*	110 (67%)	20 (67%)	0 (0%)	1 (3%)
*CTF1*	2 (1%)	2 (7%)	0 (0%)	1 (3%)
*LPL*	22 (14%)	6 (20%)	16 (10%)	4 (13%)
*TYW1B*	122 (75%)	25 (83%)	56 (34%)	12 (40%)
*KLHL8*	3 (2%)	1 (3%)	1 (1%)	2 (7%)
*PEPD*	133 (82%)	24 (80%)	28 (17%)	7 (23%)
*APOE*	90 (55%)	20 (67%)	22 (14%)	6 (20%)
*MAP3K1*	153 (94%)	25 (83%)	5 (3%)	3 (10%)
*NAT2*	134 (82%)	30 (100%)	9 (6%)	4 (13%)
*LMF1*	141 (87%)	25 (83%)	10 (6%)	5 (17%)
*COBLL1*	52 (32%)	15 (50%)	17 (10%)	7 (23%)
*JMJD1C*	151 (93%)	30 (100%)	21 (13%)	9 (30%)
*APOB*	144 (88%)	30 (100%)	111 (68%)	30 (100%)

^a^All types of variants, including benign DNA changes, SNPs, and pathogenic mutations

^b^Only pathogenic mutations

^c^Number of patients carrying variants (percentage)

**Fig. 1:**
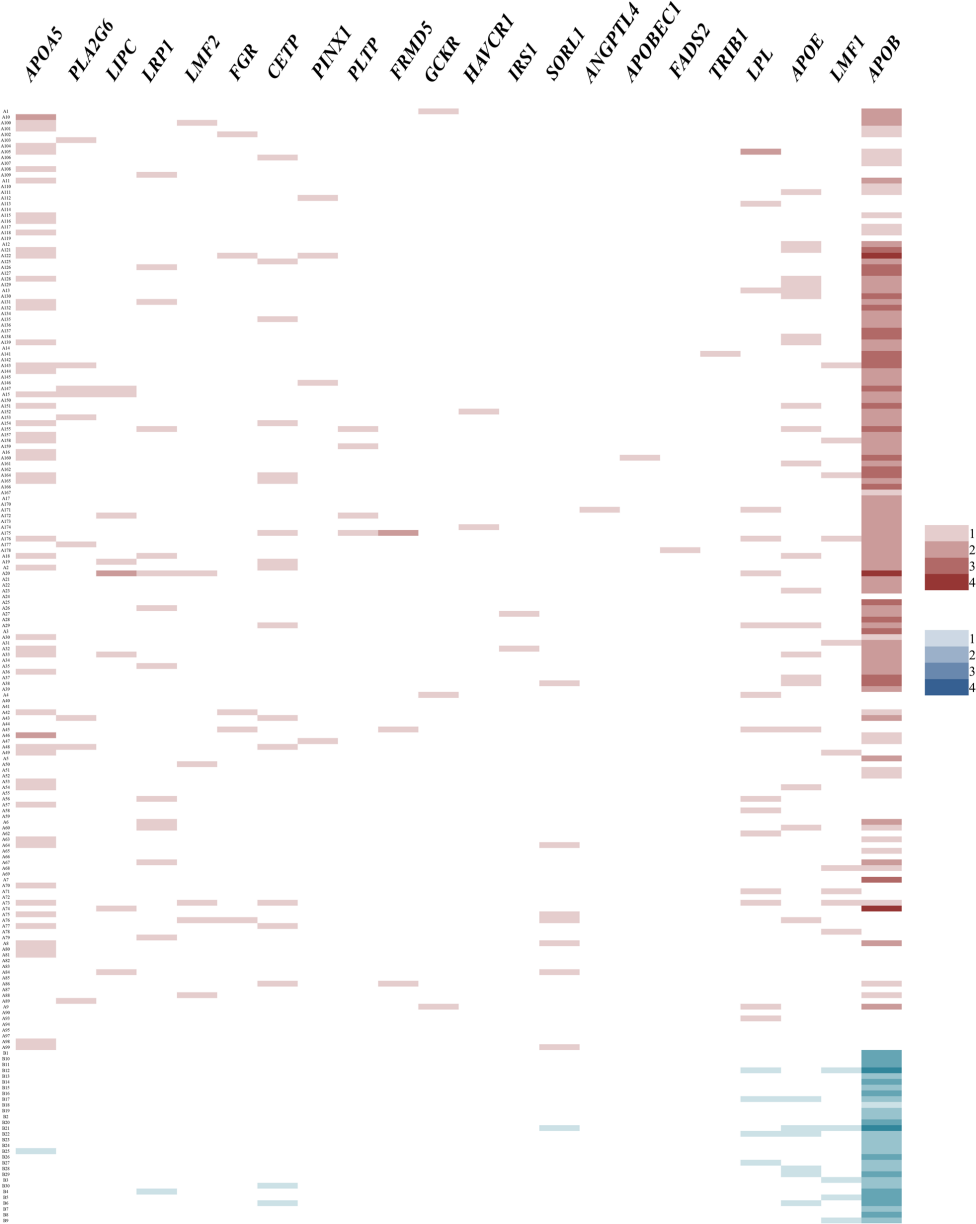
Heat map of the number of pathogenic mutations carried by 163 HLAP patients and 30 BAP patients. The Indian red squares indicate HLAP patients, and the cornflower blue squares indicate the BAP patients. The rightmost numbers “1–4” indicate the number of mutations

The results showed that pathogenic mutations in 26 genes, from *APOA5* to *TIMD4*, were more prevalent in HLAP patients than in BAP patients, with *APOA5* mutations being the most common. Figure 1 shows a heat map of pathogenic mutations in partial candidate genes carried by patients with HLAP (Indian red squares) and BAP (cornflower blue squares); the deeper the color of the square, the more mutations in this gene are carried by that patient. Patients A10 and A46 in the HLAP group harbor two different *APOA5* pathogenic mutations (Fig. 1), patient A10 harbors p.G185C, p.S35N, and patient A46 harbors p.R223C, p.G185C. However, the pathogenic mutations of *APOAB* seem to be more common in the BAP group.

#### Comparison between the mutations of *APOA5*

**Fig. 2:**
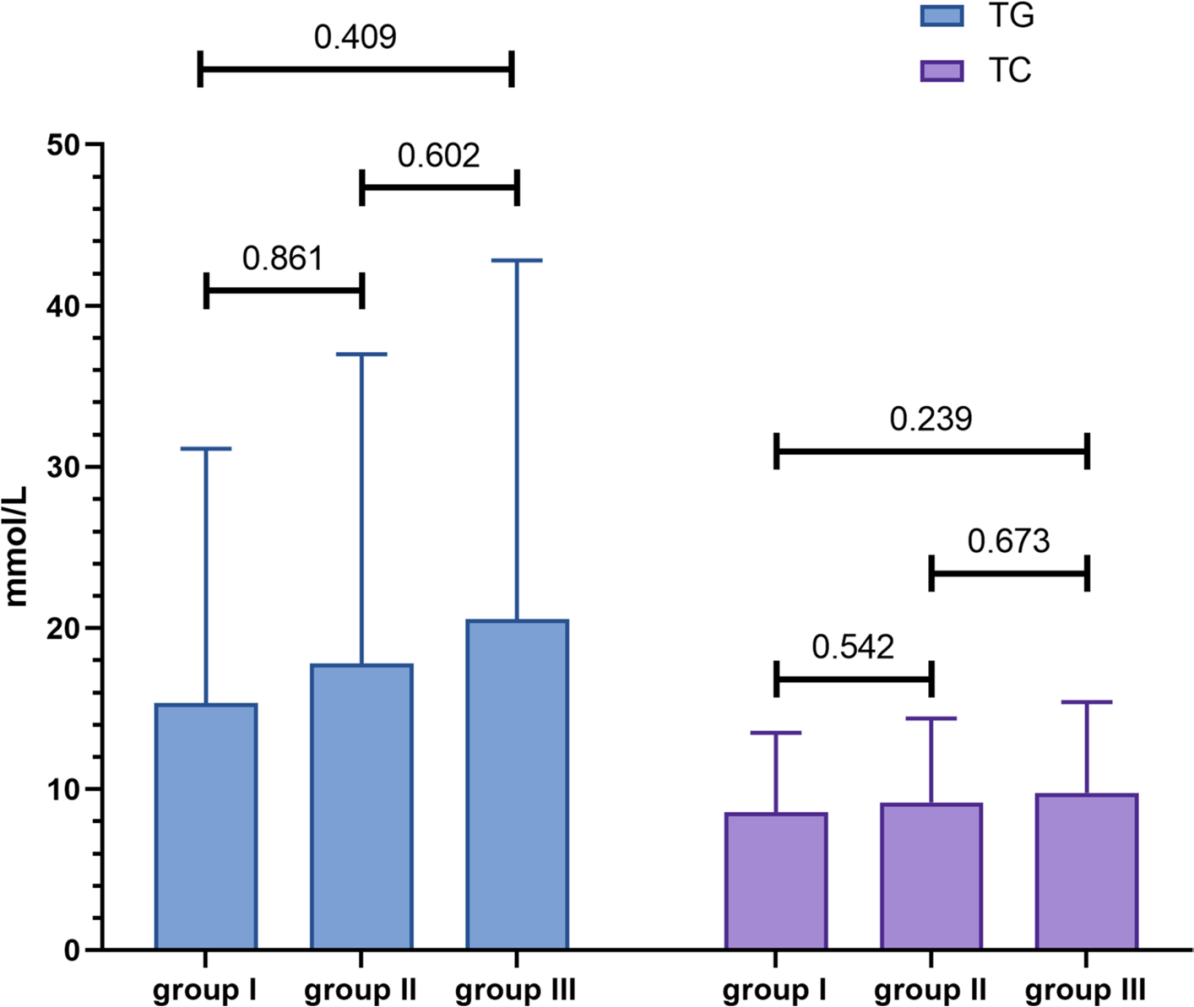
Comparison of TG and TC between patients carrying the *APOA5* mutation and other patients. Group I: patients with HLAP harboring pathogenic mutations in *APOA5*; group II: patients with HLAP harboring pathogenic mutations in 25 genes from *PLA2G6* to *TIMD4*; group III: the remaining HLAP patients

To characterize the clinical features of patients with HLAP carrying pathogenic mutations in *APOA5*, these 52 patients were taken as group I (detailed in Table 2). The 25 other genes with pathogenic mutations (ranging from *PLA2G6* to *TIMD4*) were also more common in patients with HLAP, and patients carrying these mutations were categorized in group II. The remaining patients with HLAP were allocated to Group III. The clinical characteristics of these three groups of patients are compared in Table 3. There was no significant difference in serum TG and TC levels in the three groups (see Fig. 2). Although group II had a higher CRP than group I, there was no difference in the number of MSAP and SAP in these three groups. There were more patients with DM and higher GLU levels in Group II, when compared with group I. However, patients with HLAP in group I seemed to be more prone to recurrence than those in group II (24, 46% *vs.* 13, 26%, *P* = 0.034).

**Table 3:** Clinical characteristics of patients carrying the *APOA5* mutation and others

	**group I (*n* = 52)**	**group II (*n* = 50)**	**group III (*n* = 61)**	***P***^**a**^	***P***^**b**^	***P***^**c**^
Age (years)	39.96 ± 8.40	39.98 ± 9.18	39.43 ± 11.54	0.992	0.782	0.784
Male, *n* (%)	41, 79%	39, 78%	52, 85%	0.917	0.374	0.323
Hospital stay (days)	9.65 ± 5.52	11.58 ± 10.23	10.26 ± 5.62	0.24	0.274	0.796
BMI (kg/m^2^)	27.44 ± 3.65	27.72 ± 3.17	27.60 ± 4.05	0.714	0.838	0.88
WBC (× 10ꝰ /L)	14.06 ± 3.78	14.25 ± 4.66	14.13 ± 4.14	0.968	0.933	0.902
CRP (mg/L)	153.94 ± 115.24	210.73 ± 111.22	194.64 ± 114.55	0.024^*^	0.06	0.506
BUN (mmol/L)	5.40 ± 5.39	7.39 ± 15.47	4.75 ± 2.00	0.438	0.519	0.831
Serum creatinine (μmol/L)	66.98 ± 18.05	71.73 ± 35.03	71.27 ± 26.54	0.94	0.534	0.604
Ca^2+^ (mmol/L)	2.05 ± 0.25	2.05 ± 0.24	2.07 ± 0.22	0.828	0.968	0.939
GLU (mmol/L)	9.29 ± 4.49	11.48 ± 4.87	11.20 ± 5.82	0.021^*^	0.057	0.518
TG (mmol/L)	15.33 ± 15.80	17.80 ± 19.20	20.55 ± 22.26	0.861	0.409	0.602
TC (mmol/L)	8.57 ± 4.92	9.17 ± 5.21	9.76 ± 5.63	0.542	0.239	0.673
Diabetes mellitus,* n* (%)	18, 35%	30, 60%	39, 64%	0.01^*^	0.002^**^	0.671
Hypertension, *n* (%)	13, 25%	11, 22%	12, 20%	0.721	0.496	0.763
Fatty liver, *n* (%)	43, 83%	45, 90%	50, 82%	0.284	0.92	0.231
ARDS, number, *n* (%)	2, 4%	6, 12%	2, 3%	0.156	1.000	0.137
SAP number, *n* (%)	2, 4%	6, 12%	2, 3%	0.156	1.000	0.137
MSAP & SAP number, *n* (%)	29, 56%	26, 52%	28, 46%	0.703	0.296	0.522
CTSI > 4 score number, *n* (%)	18, 35%	26, 52%	28, 46%	0.076	0.224	0.522
Recurrence, *n* (%)	32, 62%	26, 52%	30, 49%	0.331	0.188	0.768
Repeated recurrence, *n* (%)	24, 46%	13, 26%	23, 38%	0.034^*^	0.364	0.19

Group I: patients with HLAP harboring pathogenic mutations in *APOA5*, group II: patients with HLAP harboring pathogenic mutations in 25 genes from *PLA2G6* to *TIMD4*, group III: the remaining HLAP patients

*BMI* body mass index, *WBC* white blood cells, *CRP* C-reactive protein, *BUN* blood urea nitrogen, *Ca*^*2*+^ serum calcium, *GLU* blood glucose, *TG* serum triglyceride, *TC* serum cholesterol, *ARDS* acute respiratory distress syndrome, *MSAP* moderately severe acute pancreatitis, *SAP* severe acute pancreatitis, *CTSI* computed tomography severity index, Repeated recurrence: at least two episodes of recurrence in one patient

^*^P < 0.05

^**^P < 0.05

^a^*P* value of group I compared with group II

^b^*P* value of group I compared with group III, and

^c^*P* value of group II compared with group III.

A total of 97 HLAP patients and 13 BAP patients carried nine nonsynonymous SNVs of the *APOA5* gene, of which three have not been reported previously (c.563A > T, c.667C > T, and c.788G > A). Table 4 shows specific information about the pathogenic and benign variants of *APOA5*, such as the ID of the mutation in the dbSNP, the results of six bioinformatics prediction programs predicting its pathogenicity, the allele frequency of the locus, the conservation of this residue, as well as the number of patients with HLAP and BAP harboring the mutation and the mean TG. Five pathogenic SNVs (c.104G > A, c.500A > T, c.553G > T, c.563A > T, and c.667C > T) and a new frameshift insertion mutation (c.544_545 ins GGTGC) were identified in 52 patients with HLAP and 1 with BAP. Among them, 42 HLAP patients carried the c.553G > T heterozygous mutation (mean TG: 15.43 mmol/L), and 7 HLAP patients carried its homozygous mutation (mean TG: 10.77 mmol/L). The only patient with BAP who carried the c.553G > T heterozygous mutation had a TG of 1.06 mmol/L. The four pathogenic SNVs (p.S35N, p.D167V, p.K188I, p.R223C) and p.H182fs were all relatively rare (allele frequency less than 0.0001), appearing in five HLAP patients with relatively high TG levels within 48 h of onset. The p.K188I, p.R223C, p.G263D, and p.H182fs mutations are not included in various databases such as the 1000 Genomes Project, the Exome Aggregation Consortium (ExAC), and gnomAD, as well as have not been reported in other papers, so they are newly discovered mutations in this study. The phylo-HMM model predicts the conservation of residues in 20 mammals, with high scores for both D167 and R223, which are relatively well conserved.

**Table 4:** The detailed database information on *APOA5* variants

*APOA5* Variant																	HLAP group		BAP group	
Gene location	Nucleotide change	Protein change	SNP ^**a**^	variant	SIFT^**b**^	Polyphen2-HDIV^**c**^	LRT^**d**^	Mutation Taster^**e**^	Mutation Assessor^**f**^	CADD^**g**^	GnomAD-ALL^**h**^	GnomAD-EAS^**i**^	GnomAD-AMR^**j**^	phylo-HMM^**k**^	References	status	N	TG	N	TG
**pathogenic mutations**																				
exon2	c.104G > A	p.S35N	*rs*184390502	known	D	P	N	N	M	21	5.35E-05	6.69E-04	0.00E + 00	0.046		het	1	29.83	0	na
exon3	c.500A > T	p.D167V	*rs*762999453	known	D	B	N	D	M	20.9	4.00E-06	5.45E-05	0.00E + 00	0.419		het	1	21.8	0	na
exon3	c.553G > T	p.G185C	*rs*2075291	known	D	D	N	N	M	24.5	6.23E-03	6.77E-02	2.83E-04	-0.055	[39]	het	42	15.43 ± 16.89	1	1.06
																hom	7	10.77 ± 8.31	0	na
exon3	c.563A > T	p.K188I	na	novel	D	P	N	N	M	22.2	na	na	na	-0.033		het	1	13.42	0	na
exon3	c.667C > T	p.R223C	na	novel	D	D	D	D	M	29	na	na	na	0.419		het	1	16.75	0	na
exon3	c.544_545 insGGTGC	p.H182fs	na	novel	na	na	na	na	na	na	na	na	na	na		het	1	9.56	0	na
**Benign mutations**																				
exon2	c.77G > T	p.G26V	*rs*548745995	known	T	P	N	D	M	24	4.15E-06	5.62E-05	0.00E + 00	0.514		het	0	na	1	0.74
exon3	c.457G > A	p.V153M	*rs*3135507	known	T	B	N	P	L	13.66	5.09E-02	1.19E-01	4.59E-02	0.43	[40]	het	47	20.32 ± 19.55	10	0.88 ± 0.24
																hom	5	19.18 ± 22.99	1	0.71
exon3	c.538G > C	p.V180L	*rs*753800578	known	T	B	N	N	M	17.37	2.41E-05	3.29E-04	0.00E + 00	-0.042	[41]	het	0	na	1	0.81
exon3	c.788G > A	p.G263D	na	novel	T	B	N	N	L	1.838	na	na	na	-0.042		het	1	108.75	0	na

*het* heterozygote, *hom*: homozygote, *N* number of people carrying the mutation in the group

^a^The number of mutations in the dbSNP (v147), *na *not available

^b^SIFT, *D*: deleterious, *T*: tolerated

^c^Polyphen2-HDIV, *D *probably damaging, *P *possibly damaging, *B *benign

^d^LRT, *D*: deleterious, *N *neutral, *U *unknown

^e^Mutation Taster, *D *disease causing, *N *polymorphism, *P *polymorphism automatic

^f^Mutation Assessor, high (*H*) and medium (*M*) are damaging, and low (*L*) and neutral (*N*) are benign

^g^CADD > 15 is deleterious, ≥ 20 is anticipated to be ranked among the top 1% of the most deleterious mutations in the human genome

^h^^−^^j^The allele frequency (%) in total / East Asian / Latin American populations from gnomAD

^k^The phylo-HMM model assesses the conservation of residues in 20 mammalian species; the higher the score, the greater the conservation

**Fig. 3:**
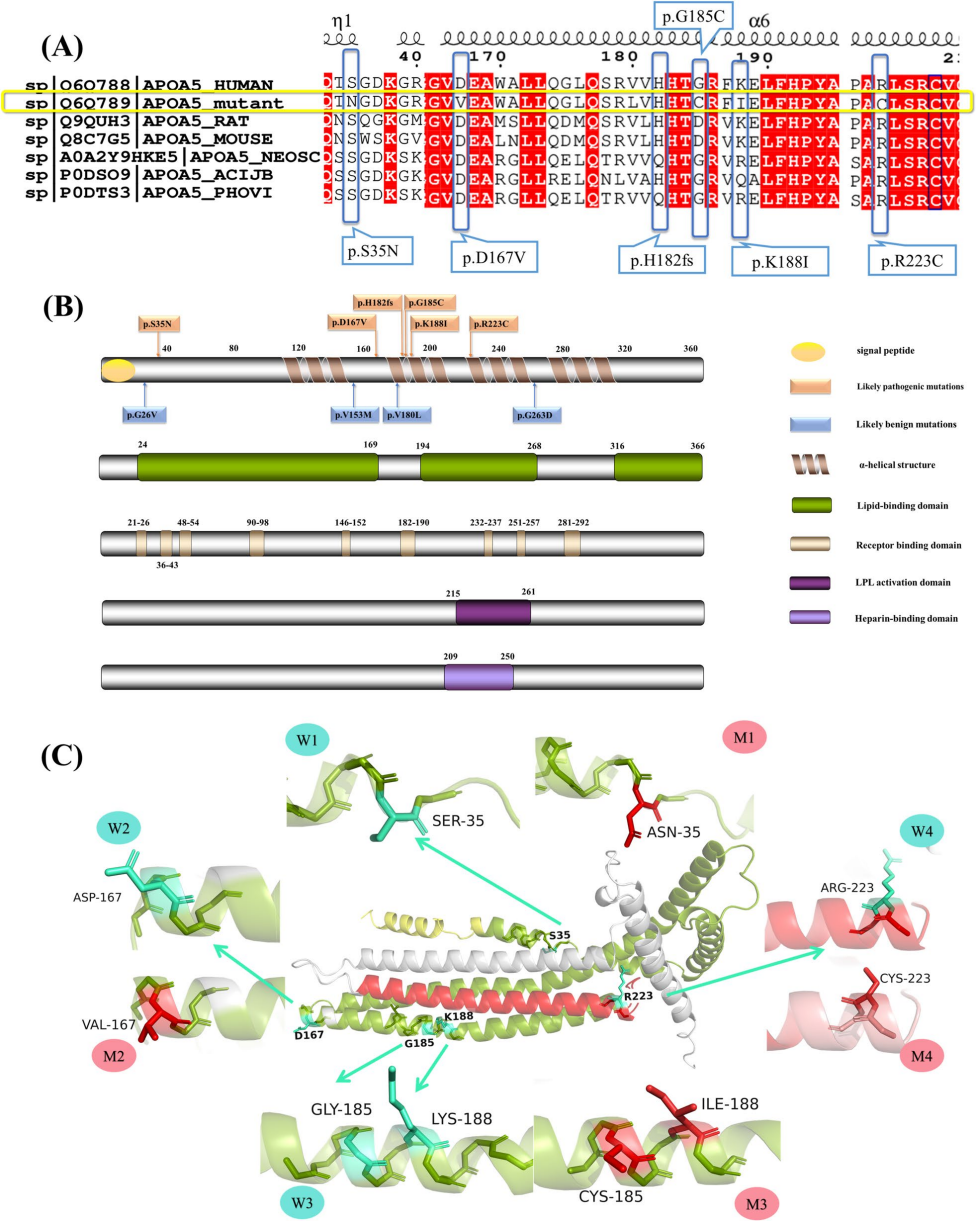
apoA-V multiple sequence alignment and schematic structure. **A** Sequence alignment of apoA-V from multiple species, from top to bottom: human (HUMAN) wild-type apoA-V, mutated human apoA-V, rat (RAT), mouse (MOUSE), Hawaiian monk seal (NEOSC), cheetah (ACIJB), and harbor seal (PHOVI). **B** Schematic diagram of apoA-V showing its main domains and *APOA5* mutations. Signal peptide (Met1–Ala23). α-Helical structures (Ala112–Val153, Leu173–Val213, Pro221–Ala262, Gln275–Leu314). Lipid binding domains (Arg24–Val169, Tyr194–Gly268, Pro316–Pro366). Receptor binding domains (Thr21–Gly26, Gly36–Gln43, Lys48–Ala54, Val90–Glu98, Glu146–Arg152, His182–Leu190, Ser232–Leu237, Asp251–Leu257, Val281–Thr292). Heparin binding domain (Leu209–Leu250). LPL activation domain (Pro215–Phe261). **C** Predicted tertiary structure of wild-type apoA-V and pathogenic mutations. The structure of wild-type apoA-V is depicted in the center. The yellow part represents the signal peptide, the green part is the lipid binding domain, the red part is the LPL activation domain. W1–4 indicates wild-type 1–4. Mutations 1–4 (M1–4) represents the predicted tertiary structure of p.S35N, p.D167V, p.G185C, p.K188I, p.R223C

Figure 3(A) illustrates the alignment of sequences of apoA-V from several mammals, along with pathogenic mutations identified in this study, and it can be seen that residues S35, D167, and R223 are strictly conserved. Patient A10 (carrying p.S35N) suffered five episodes of HLAP, and patient A15 (p.D167V) had three episodes, and their TG on day 1 from onset was 29.83 and 21.8 mmol/L, respectively. Patient A46 (carrying p.R223C) was admitted to the hospital on day 2 of illness when the TG was 16.75 mmol/L. All six software programs assessed p.R223C as pathogenic, with a CADD score of 29 (a mutation is among the top 1% of the most pathogenic mutations in the human genome when CADD ≥ 20). Among the benign changes, p.G26V and p.V180L appeared in patients with BAP with TGs of 0.74 and 0.81 mmol/L on day 1, respectively. Previous studies have shown that p.V153M has no significant effect on TG [14].

Currently, the high-level structure of apoA-V has not been clarified. Data from SignalP-5.0, InterPro, SMART, and previous studies [42–46] were combined to form several important domains of wild-type apoA-V (Fig. 3B), which are closely related to its lipid-lowering function. Its tertiary structure was simulated with AlphaFold, and the mutated amino acids were modified with PyMOL to clearly demonstrate the residue alterations (Fig. 3C). All pathogenic *APOA5* mutations were found to occur in the essential domains of apoA-V (Fig. 3B).

The p.D167V and p.S35N mutations are located in the first lipid-binding domain of apoA-V (Arg24–Val169), and p.R223C is located in another lipid-binding domain (Tyr194–Glu268). They may affect an important function of apoA-V, which is the binding of lipids (Fig. 3B). With regard to p.D167V, when residue Asp167 is mutated to Val167 (Fig. 3C), the hydrophilic and negatively charged aspartate is replaced by the hydrophobic and uncharged valine, and the polarity and charge of the residue are changed.

Except for p.S35N, the polarity and / or charge of the residue have always been changed in pathogenic mutations. Given that both Ser and Asn are polar and uncharged amino acids, there is no change in amino acid polarity or charge in p.S35N. However, the Ser35 residue is considered to be a strictly conserved sequence in various organisms. The predictions for p.S35N by SIFT, Polyhen2-HDIV, Mutation Assessor, and CADD all turned out to be pathogenic.

In p.R223C, the basic and positively charged Arg becomes the uncharged Cys. Residue Arg223 is also located in the lipoprotein lipase (LPL)-binding domain, which is utilized by apoA-V for binding to LPL to enhance lipid-lowering effects. The p.R223C not only results in a decrease in the positive charge of residue 223, which impairs the ability of apoA-V to bind lipids, but also may impair its ability to bind to LPL and heparin (Fig. 3B).

The receptor-binding domain (RBD) of apoA-V is also related to its lipid-lowering function; p.H182fs (c.544_545 insGGTGC), p.G185C, and p.K188I appeared in the sixth RBD (His182–Leu190). With the insertion of GGTGC between bases 544 and 545, the amino acids were altered, and TG on day 2 was 9.56 mmol/L in patient A30 carrying p.H182fs. In the p.G185C mutation, hydrophobic Gly is replaced by hydrophilic Cys. In the p.K188I mutation, hydrophilic and positively charged Lys is mutated to hydrophobic and uncharged Ile. The altered hydrophobicity and lost positive charge of the residues may weaken the binding of RBD to other proteins.

Among benign changes, p.G26V, p.V153M, and p.V180L did not change the polarity or charge of the residues. Although there are changes in polarity and charge caused by p.G263D, residue G263 is extremely poorly conserved (phylo-HMM: –0.042), and all six software programs consider p.G263D to be non-pathogenic.

## Discussion

In this study, whole exome sequencing results from 163 HLAP patients and 30 BAP patients were studied to investigate which genes are clinically significant for severe HTG in patients with HLAP.

As mentioned earlier, genetic mutations are the primary etiology of severe HTG, with the underlying disease being a secondary etiology [8–10]. Although the HLAP group had a slightly higher BMI, the BMI of the BAP group also met the diagnostic criteria for overweight (mean: 25.11 kg/m^2^). However, the mean TG of the 19 overweight patients with BAP (27.74 kg/m^2^) was only 0.96 mmol/L. Among 18 HLAP patients with a normal BMI (22.02 kg/m^2^), their mean TG was up to 22.62 mmol/L. It can be seen that there is no inevitable relationship between obesity and HTG. Similarly, the mean TG of the four BAP patients with diabetes was 0.755 mmol/L, whereas the 91 non-diabetic patients with HLAP had a mean TG of 22.86 mmol/L.

More than one type of software was used to interpret variants, in accordance with the recommendations of the American College of Medical Genetics and Genomics (ACMG) [47]. The preliminary screening scheme for potentially pathogenic mutations was first to enroll all mutations that were evaluated to be “deleterious” by SIFT, a classical prediction software with high sensitivity and low specificity [48]. The outcomes were then combined with results from one of the five commonly used prediction software programs (PolyPhen, LRT, Mutation Taster, Mutation Assessor, and CADD) to improve the specificity of the scheme. A comprehensive assessment was performed by combining the specific location of the mutation in the protein, residue conservatism, changes in the properties of the residue, and clinical information on the carrier, which is consistent with the ACMG’s recommendations.

In this study, a total of 26 genes from *APOA5* to *TIMD4* were found to be more likely to develop pathogenic mutations in HLAP patients (Table 2), among which the *APOA5* pathogenic mutations (HLAP 32% *vs.* BAP 3%) were the most striking (Figure 1). However, in terms of TG, AP severity, and comorbidities, there was no significant difference between the HLAP patients with *APOA5* pathogenic mutations and other HLAP patients without *APOA5* pathogenic mutations (detailed in Table 3).

Six pathogenic mutations in *APOA5* were identified in this study (Table 4), including five rare mutations (p.S35N, p.D167V, p.K188I, p.R223C, and p.H182fs), whose carriers had chylemia on the day of admission. Among them, p.S35N and p.D167V, documented in the gnomAD, are more common in East Asia than in other regions. The patient harboring p.S35N was admitted for his fifth HLAP with a TG of 29.83 mmol/L on the first day of onset. In addition, p.K188I (c.563A>T), p.R223C (c.667C>T), and p.H182fs (c.544_545 insGGTGC) were newly identified in this study.

Among the pathogenic mutations of *APOA5*, c.553G>T (Gly185Cys) appeared most frequently, with 42 HLAP patients (25.77%, heterozygous), 7 HLAP patients (4.29%, homozygous), and 1 BAP patient (3.33%, heterozygous). 72% (38 / 53) of patients who carried pathogenic mutations in *APOA5* were born in Jiangsu province, China, and 100% (7 / 7) of the patients carrying *APOA5* p.G185C homozygous mutations clustered in Suzhou, Jiangsu province. There was no consanguinity among them; it is speculated that an ancestry effect may have been involved. The only individual in the BAP group who carried the p.G185C heterozygous mutation was patient B25, who was 83 years old at the time of onset and had a light and predominantly vegetarian diet. During hospitalization, his lipid profile remained normal (TG maxima of 1.60 mmol/L and TC maxima of 5.16 mmol/L). He had six offspring, four of whom have been diagnosed with hypertriglyceridemia. Therefore, this patient was advised to add a lipid profile to his annual physical examination.

The apoA-V consists of 366 amino acid residues [12], and its secondary structure is not yet fully understood. The signal peptide cleavage site was predicted to be between A23 and R24 using SignalP-5.0 (probability: 0.8135). When this is combined with the prediction results of InterPro, Met1 to Ala23 form the signal peptide. The remaining 343 residues became the mature apoA-V, 50% of which formed α-helical structures [44]. When each residue’s confidence score is > 70 (pLDDT > 70) according to AlphaFold prediction, residues Ala112–Val153, Leu173–Val213, Pro221–Ala262, and Gln275–Leu314 are predicted to form helical structures. Meanwhile, InterPro and SMART were used to predict α-helical formations, yielding results of Thr236–Glu256 and Leu235–Ala260, which were included in the third helix of AlphaFold (Pro221–Ala262). Therefore, the prediction results of AlphaFold were adopted in this study (Fig. 3B).

Currently, the most widely accepted lipid-lowering mechanism of apoA-V is its stimulatory effect on LPL [49]. Merkel *et al.* proposed the definition of an LPL-HSPG (heparan sulfate proteoglycan) complex, indicating that apoA-V had a stabilizing effect on it, thereby promoting the lipolytic function of LPL [50]. The Pro215–Phe261 located in the full-length apoA-V had an LPL-activating function, while Leu209–Leu250 carried a strong positive charge and may interact with endothelial surface HSPGs [42, 43]. R223 is located in a unique position where the α-helix, lipid-binding domain, LPL-binding domain, and heparin-binding domains overlap. The positive charge of residue 223 is reduced when polar Arg becomes nonpolar Cys (Fig. 3C). Therefore, it has been speculated that p.R223C might not only affect the binding of apoA-V to lipids and heparin but also impair the activity of LPL. R223 is position-specific and highly conserved (Fig. 3A). A HLAP patient A46 carrying this mutation was admitted to the hospital on day 2 after onset with a fasting TG of 16.75 mmol/L and chylemia. p.R223C was predicted to be pathogenic by all six bioinformatics prediction programs (PolyPhen, LRT, Mutation Taster, Mutation Assessor, and CADD).

From 2003 to 2009, several studies found that Arg24–Ala169, Tyr194–Glu268 and the C-terminus of full-length apoA-V had lipid-binding ability [44–46]. The three pathogenic mutations (p.S35N, p.D167V, and p.R223C) presented in these domains may affect the binding of apoA-V to lipids, resulting in abnormally elevated plasma TG in carriers. Residue Ser35 is strictly conserved in multiple animals (Fig. 3A), and patients carrying p.S35N had TG of 29.83 mmol/L on day 1 with significant chylemia; it was predicted to be pathogenic by four software programs. Asp167 is strongly conserved (phylo-HMM: 0.419), and both the hydrophilicity and negative charge of this residue are lost when Asp is mutated to Val (p.D167V).

Although p.G26V and p.V153M also appeared in the lipid-binding domain, neither the charge nor the hydrophobicity of these residues were altered, and the patients carrying p.G26V had TG in the normal range (0.74 mmol/L). Residue G263 appears at the C-terminus of apoA-V, but it is extremely poorly conserved in mammals (phylo-HMM: –0.042), and p.G263D was predicted to be benign by all six software programs.

A receptor-mediated mechanism is another hypothesis for the lipid-lowering effect of apoA-V. It has multiple domains that interact with members of the LDL receptor gene family (e.g., LDLR, LRP1, SorLA / LR11, and sortilin) [51]. Biosiris was used by Talmud [52] to predict the nine potential receptor-binding domains (RBDs) carrying positive charges that are likely to be engaged in protein-protein interactions. The p.G185C, p.K188I, and p.H182fs (c.544_545 insGGTGC) located in the helical region also appeared in the sixth RBD (H182-L190) (Fig. 3B). When Lys188 was replaced by Ile188 (Fig. 3C), both the hydrophobicity and positive charge of residue 188 were weakened, which affected the binding of RBD to proteins. The other two lipid-lowering theories of apoA-V speculate that it may reduce the secretion of VLDL and apoC-III in the liver [53, 54], which remains to be confirmed.

Taken together, all the above pathogenic mutations were located in the key domains of apoA-V, which can affect the function of apoA-V by altering the properties of the residues and increasing the risk of severe HTG in carriers.

### Strengths and limitations of the study

The effect of pathogenic gene mutations on severe HTG in patients with HLAP was first explored in this study, especially the APOA5 gene mutations. By combining multiple bioinformatics sources, several pathogenic mutations have been shown to be highly likely to affect TG. These findings broadened the current understanding of genes related to lipid metabolism and will contribute to future etiological diagnosis and locus-specific therapy.

The shortcomings must be acknowledged as follows: there is a minority of patients with HLAP (6.1%, 10 / 163) who do not carry pathogenic mutations in the 53 lipid-related genes mentioned above. In addition, owing to the lack of experimental equipment, the activity of apoA-V in serum was unable to be quantified and measured in this study and needs to be further investigated.

## Conclusions

In this study, the pathogenic mutations of *APOA5* were identified by performing whole exome sequencing in patients with HLAP and BAP. The positive correlation between multiple pathogenic mutations in *APOA5* and severe HTG was confirmed. Three unreported SNVs and a frameshift insertion mutation were also identified to expand the *APOA5* gene profile. The effects of these pathogenic mutations were further clarified by a multifaceted analysis using clinical information, software calculations, database information, and residue conservation. These findings will contribute to etiologic diagnosis and precision medicine for HLAP patients with severe HTG, which may eventually prevent patients from suffering recurrent HLAP.

## Data Availability

Reference genome (GRCh37.p13) download link: https://ftp.ncbi.nlm.nih.gov/genomes/all/GCF/000/001/405/GCF_000001405.25_GRCh37.p13/GCF_000001405.25_GRCh37.p13_genomic.fna.gz. The remaining datasets generated during and/or analyzed during the current study are available from the corresponding author on reasonable request.

## Abbreviations

TG: Serum triglyceride
HTG: Hypertriglyceridemia
AP: Acute pancreatitis
HLAP: Hyperlipidemic acute pancreatitis
BAP: Biliary acute pancreatitis
*APOA5*: Apolipoprotein A5
apoA-V: Apolipoprotein A-V
CM: Celiac microsomes
VLDL: Very low-density lipoproteins
HDL: High-density lipoproteins
WBC: White blood cells
CRP: C-reactive protein
BUN: Blood urea nitrogen
Ca^2+^: Serum calcium
GLU: Blood glucose
TC: Serum cholesterol
MAP: Mild acute pancreatitis
MSAP: Moderately severe acute pancreatitis
SAP: Severe acute pancreatitis
SNP: Single nucleotide polymorphism
SNV: Single nucleotide variant
InDel: Insertion and deletion
het: Heterozygote
hom: Homozygote
BMI: Body mass index
ARDS: Acute respiratory distress syndrome
DM: Diabetes mellitus
LPL: Lipoprotein lipase
RBD: Receptor-binding domain
HSPG: Heparan sulfate proteoglycan
